# Using non-human primates to benefit humans: research and organ transplantation—response to César Palacios-González

**DOI:** 10.1007/s11019-015-9676-z

**Published:** 2015-12-08

**Authors:** Wybo Dondorp, David Shaw, Guido de Wert

**Affiliations:** Department of Health Ethics and Society, Maastricht University, PO Box 616, 6200 MD Maastricht, The Netherlands; Institute for Biomedical Ethics, University of Basel, Basel, Switzerland

We thank César Palacios-González for responding to our paper on “The use of non-human primates to benefit humans: research and organ transplantation”.^1^ In our paper we describe how emerging biotechnology may soon allow the creation of genetically human organs inside animals. Those organs would grow from human induced pluripotent stem cells injected into embryos of host animals genetically modified so as to block the growth of the relevant animal organ. This procedure would result in the birth of a chimeric animal that may have human cells contributing to any of its tissues, but with the target organ being fully human. Although scientists working on this scenario expect that pigs might be a suitable species, it is not impossible that the technology would only work with non-human primates. In our paper we considered whether it would be ethically acceptable to use non-human primates for this purpose, using the widely endorsed consensus about the acceptability of using non-human primates in medical research as a benchmark. If, under conditions of proportionality and subsidiarity, non-human primates may be used for research, we think the same conclusion would hold for using them as a source of human transplantation organs. However, we made the following qualification with regard to the so-called great apes: “to the extent that primates actually meet the criteria for personhood, they should be treated as persons rather than animals, making the proportionality and subsidiarity principles irrelevant. For the purposes of this paper, we will assume that the current consensus position is correct, and primates (with the possible exception of great apes) are not persons”. Given that we have thus bracketed out the great apes from our consideration of the ethics of using non-human primates as a potential new source of transplantation organs, we found it interesting to note that Palacios-González has read us as precisely trying to make a case for using great apes for this purpose. As his critique builds on a misrepresentation of what our paper was about, his whole battery of claims purportedly showing “that their arguments fail” is off target.

Firstly, Palacios-González says that we propose to use the principles of proportionality and subsidiarity to justify the use either for research or for organs of creatures who might perhaps qualify as persons (great apes). That would be a highly problematic endeavor indeed, precisely because, as we say in the above quotation from our paper, those principles do not apply in cases where we are dealing with persons. We have not tried to argue that great apes might be used in research or as a source of organs. Instead, we have argued that non-human primates may be used for those purposes, while bracketing out the great apes, precisely because they are in the borderline area of personhood.

Secondly, Palacios-González says we have failed to acknowledge that great apes may be closer to personhood than some humans. Ignoring this, we would have failed to grasp the implication “that it would be unjustifiable to sacrifice great-ape/human chimeras for the sake of human borderline persons, contrary to what Shaw et al. endorsed”. However, we did not endorse this, nor is it an implication of anything we have argued for.

Thirdly, Palacios-González maintains we have “ignored the ethics literature on borderline persons”. Again, because we did not try to defend the use of great apes, there was no need to discuss the status of borderline persons in our paper.

Fourthly, according to Palacios-González, we presented a “deeply incorrect version of McMahan’s moral theory” by quoting him out of context. In the lines we quoted from Jeff McMahan, he argues that animals “have a weaker time-relative interest in the continuation to live than a person normally does”. We only used this as a further illustration of different accounts of why harming animals would be less objectionable than harming people, underlining that because of its sliding scale, the time-relative interest account fares no better than others in telling “whether the use for research of animals with slightly less psychological capacity than humans is justified” (or indeed, whether the use of human borderline persons would be justified). Clearly, this is precisely why McMahan insists that a complete account of the morality of killing needs a ‘threshold of respect’ as a second tier. But we were not in the business of trying to explain McMahan’s theory, nor did we intend to use that theory to build (what Palacios-González mistakenly takes to be) our own position. We do not see how we have misrepresented McMahan, or why we should have quoted him more extensively.

We agree with Palacios-González that any attempt to justify “the killing [of] human/great ape chimeras for their organs” would face important difficulties given the unclear status of those creatures who/that may or may not qualify as persons. As said, this is precisely why we have limited our paper to the far more circumscribed issue of whether it would be justified to use non-human primates for organ creation, focusing on the category about which there is a consensus that they may under conditions be used for research and leaving the great apes aside. So how can Palacios-González have so seriously misread our paper? It seems he has built his interpretation on two formulations in our paper that are, in hindsight, unfortunate.

One is in the last few sentences of the section on Proportionality (fourth page, upper part right column). Here we say that because great apes may be more appropriate donors, the question arises “whether the distinction between lesser and great apes [as made in the context of research] is relevant in this context”. On a less charitable interpretation one may read us as questioning whether the distinction is *morally* relevant, thus opening the door to the use of great apes. That is precisely how Palacios-González has read us. However, what we meant is that whereas the distinction is useful in the context of research, in the sense of delineating a category of non-human primates that (under conditions) could justifiably be used for the purpose, this might not hold for the context of creating chimera organs, if it is indeed the case that only great apes would be good candidates for being chimera organ donors. In that case, the conclusion would have to be that the reasoning behind the justification of using non-human primates for research cannot also serve for justifying their use as chimeric organ donors. Secondly, we should not have referred to great apes when illustrating how the justification of using non-human primates for research or organs is more problematic to the extent that the human need is lesser (one before last page, upper part left column). The fact that we used this formulation here is indeed unfortunate, because it is clearly out of tune with the main arguments in our paper. As such, it certainly does not suffice for building the case that Palacios-González brings against us.

## Footnotes

1 César Palacios-González. The ethics of killing human/great-ape chimeras for their organs: a reply to Shaw et al. Med Health Care Philos. 21 Aug. 2015.

